# 4399 Empowered Transitions: Understanding the Experience of Transitioning to Adult Care Among Adolescents with Inflammatory Bowel Disease and Their Parents Using Photovoice

**DOI:** 10.1017/cts.2020.266

**Published:** 2020-07-29

**Authors:** Jordyn Feingold, Laurie Keefer, Ksenia Gorbenko, Halley Kaye-Kauderer, Michelle Mendiolaza

**Affiliations:** 1 Icahn School of Medicine at Mount Sinai

## Abstract

OBJECTIVES/GOALS: Inflammatory bowel diseases (IBD) are most often diagnosed in adolescence and young adulthood, affecting 10 in 100,000 pediatric patients in the US and Canada. Adolescents with IBD are vulnerable to poorer outcomes and higher health costs, partially attributable to disruptions in the continuity of care in the transition from pediatric to adult care settings. There is currently no consensus among providers about the timing of initiation and completion of the transition process for adolescents and young adults with IBD, and access to structured pediatric transition readiness programs are lacking, with a paucity of research to evaluate relevant clinical outcomes in such existing programs. While prior studies have primarily examined barriers and facilitators of successful transitions from the provider perspective, only few studies have systematically examined such factors from the patient and caregiver perspective. We wish to better understand the experience of living with IBD for adolescents and young adults, as well as their parents, to understand barriers and facilitators of successful transitions in care. Ultimately, we wish to articulate best practices in this domain in order to create and evaluate a transitions program for patients and parents at the Mount Sinai IBD Center. METHODS/STUDY POPULATION: We are recruiting 15-25 patient-parent dyads to complete our study. At recruitment, we collect baseline quantitative metrics from patients pertaining to demographics, disease characteristics, transition-readiness, self-efficacy, resilience, disease-specific health knowledge, and health literacy. From parents, we collect demographic information, concordance metrics (e.g. how parents perceive their children’s resilience, self-efficacy), parenting style questionnaires, and others. These data are used to understand the characteristics of the young adults and parents within our sample to ensure that the results of our study will be generalizable to a diverse range of patients and families. We then train our patient-parent dyads in Photovoice, the primary method of our study. Photovoice is a community based participatory research (CBPR) methodology used in health education and other fields. The method employs photography for participants to capture their experiences living with IBD, or being a parent to a child with IBD. We then interview all participants about the photos using a standard script employed in Photovoice. All surveys are transcribed and coded for thematic analysis. Based on our findings, we hope to determine phenotypes of patient-parent dyads who are likely undergo successful transitions as well as those at higher risk, understand competencies necessary for successful transitions, and create a comprehensive transitions program for the IBD Center that can be applied with all patients undergoing transitions from pediatric to adult GI care. RESULTS/ANTICIPATED RESULTS: We currently have 26 patients and 25 parents (1 pair of siblings) aged 14-25 enrolled in the study. We hypothesize that adolescents with higher baseline resilience, efficacy, disease-specific health knowledge, and less active disease will have more successful transitions than adolescents with lower scores on these metrics. Similarly, we predict that adolescents with lower baseline resilience, self-efficacy, disease-specific health knowledge and more active disease will be ideal candidates for a more robust transition-readiness program. Further, we hypothesize that children of more authoritarian parents will be less prepared for transition than those with assertive parents. We are currently in the process of conducting patient/parent interviews, and have collected 6 interviews thus far. We will begin the qualitative coding process once we have four interviews from each cohort. Themes emerging thus far involve: medication management, psychiatric co-morbidity, social support, direct communication with doctors, the role of surgery, school absences, travel, and others. DISCUSSION/SIGNIFICANCE OF IMPACT: Transition-readiness is defined as a series of skills in the realms of knowledge, information gathering, self-management, and decision-making that must be mastered by a patient in preparation for a healthcare transition, such as that from pediatric to adult IBD care. It has been shown that many clinicians who rely on subjective measures such as perceived health literacy overestimate transition readiness in their IBD patients. Many pediatric gastroenterologists who use more objective measures rely on a validated self-report questionnaire, the Transition Readiness Assessment Questionnaire (TRAQ) to assess readiness for transition and to facilitate discussions around the skills necessary to transition, including appointment keeping, tracking health issues, managing medications, talking with providers, and managing daily activities. However, the TRAQ has been shown to be limited in its ability to predict transition readiness independently of age, and ignores both provider and family perspectives. Given the critical role of parents in medical decision making, and the differential emphasis of the caregiver role in pediatric versus adult IBD care paradigms, it is vitally important to identify barriers to transition as well as differences in perspectives between adolescents living with IBD and their parents. Our study is the first to employ Photovoice, a method that ‘gives a voice to the voiceless’ in the gastroenterology space, in order to understand the needs that adolescents and young adults themselves perceive as critical in promoting transition-readiness. We include parents in this inquiry in order to understand how parental perceptions of their children’s transition-readiness promote or stifle successful transitions and independent disease self-management. We will ultimately use this data to create a Transitions program to evaluate in our center for adolescents with IBD and their parents.

